# Assessment of physicochemical alterations in 3D-printed biodegradable implants under biomimetic conditions for cranial defect repair

**DOI:** 10.1007/s10544-025-00788-5

**Published:** 2026-01-07

**Authors:** Eungtae Lee, Yeonguk Seong, Jihee Jeong, Yeji Cheon, Joonho Eom, Jinhyun Kim, Sangbae Park, Jong Hoon Chung

**Affiliations:** 1 Medical Device Research Division, Pharmaceutical and Medical Device Research Department, National Institute of Food and Drug Safety Evaluation, Cheongju, 28159 Republic of Korea; 2 Department of Biosystems Engineering, Seoul National University, Seoul, 08826 Republic of Korea; 3 Integrated Major in Global Smart Farm, College of Agriculture and Life Sciences, Seoul National University, Seoul, 08826 Republic of Korea; 4 Research Institute for Agriculture and Life Sciences, Seoul National University, Seoul, 08826 Republic of Korea; 5 ELBIO Inc, Seoul, 08812 Republic of Korea

**Keywords:** Biodegradable, Additive manufacturing, Implantable medical device, Physicochemical characteristics, Simulated cranial defect physiological condition

## Abstract

Once implanted, biodegradable devices gradually deteriorate, potentially compromising clinical performance. Consequently, evaluating the alterations in physicochemical characteristics after implantation is crucial. Nonetheless, there is currently no established methodology for precisely assessing these alterations. This study sought to develop accurately simulated cranial defect physiological conditions (SCDPC) and examine the physicochemical modifications in biodegradable cranioplasty plates (BCP) to anticipate their performance changes following implantation in humans. We analyzed the physicochemical property alterations of BCP following 24 weeks of exposure to SCDPC. Following 24 weeks under SCDPC, the BCP showed a notable reduction in mass (− 0.79%) and tensile strength (− 69.30%). A decrease in molecular weight was noted after 12 weeks of implantation in rabbits (− 9.67%) and following 12 weeks of exposure to SCDPC (− 4.73%). The physicochemical alterations identified under simulated *in vitro* cranial defect conditions closely mirrored those found in the *in vivo* setting. In summary, assessing BCP under SCDPC offers an innovative and dependable approach for precisely forecasting performance shifts after implantation. This strategy could provide meaningful guidance for the advancement of BCP and various other biodegradable medical devices.

## Introduction

Cranioplasty is a surgical procedure aimed at repairing skull defects resulting from trauma or surgical interventions such as decompressive craniectomy. This procedure involves the use of various materials to reconstruct the damaged cranium, typically either a segment of the patient’s own bone or a synthetic device. Due to its excellent integration with bone, low immune reaction, and anatomical fit, autologous bone grafting is widely accepted as the standard approach in cranioplasty (He 2024). However, despite its advantages, autologous bone grafting is associated with the risk of bone resorption, a phenomenon that remains poorly understood and has been widely documented in the literature (Rienzo et al. 2024; Mee et al. 2022; Sari et al. 2017). Bone resorption can compromise the structural integrity of the graft, leading to various clinical complications, including postural headaches, facial asymmetry, and pain or discomfort along the edges of the cranioplasty site. For synthetic devices, titanium is considered a key alternative because of its superior biocompatibility, corrosion resistance, and an elastic modulus that more closely approximates that of bone compared to other metals (Neumann and Kevenhoerster 2011). In addition, the incorporation of ceramic calcium phosphates such as hydroxyapatite (HA) (Millward et al. 2022) and beta-tricalcium phosphate (β-TCP) (Park et al. 2022), along with polymers like polymethylmethacrylate (PMMA) (Msallem et al. 2022), polyetheretherketone (PEEK) (Kwarcinski et al. 2017), and polycaprolactone (PCL) (Kirmanidou et al. 2024), has expanded the range of material options for cranioplasty procedures. Such advancements have marked a new phase in cranioplasty, particularly with the integration of computer-aided design and three-dimensional (3D) printing, which allow for the fabrication of patient-specific medical devices precisely aligned with individual anatomical structures (Santis et al. 2021; Czyżewski et al. 2022; Kanwar and Vijayavenkataraman 2021).

Furthermore, 3D-printed medical devices—such as biodegradable cranioplasty plates (BCP)—have been developed using medical imaging technologies like CT, MRI, and 3D scanning to produce personalized, patient-specific implants. 3D printing enables the fabrication of intricate medical devices that are difficult to create through traditional techniques, while also supporting small-scale manufacturing of highly personalized implants. The majority of 3D-printed medical devices are made from biodegradable polymers, whose physical characteristics can be affected by the physiological environment of the implantation site as they progressively break down. Alterations in the physicochemical properties of these materials post-implantation may impair their functionality, potentially compromising clinical outcomes and hindering the device from achieving its intended purpose. Thus, accurately assessing the properties of biodegradable medical devices requires accounting for the physiological environment of the implantation site—including contact with blood and body fluids—to effectively predict and evaluate potential performance deterioration.

Although biodegradable medical devices are increasingly utilized, a standardized method that accounts for the physiological environment of the implantation site when evaluating their physicochemical characteristics-such as degradation patterns and long-term stability-is still lacking, as previously noted by our research group (Lee et al. 2025). Current studies primarily evaluate degradation using research scaffolds in buffer solutions, such as phosphate buffer solution (PBS), rather than under conditions that replicate the specific factors of the implantation site, such as composition and pH. This limitation hinders the accurate prediction of post-implant performance, raising concerns regarding the clinical reliability of biodegradable medical devices. To address this issue, the development of standardized evaluation methods that simulate physiological conditions is essential for assessing physicochemical changes and ensuring the long-term functionality and safety of these devices. There is a lack of research assessing *in vitro* changes in BCP properties after exposure to simulated cranial defect physiological conditions (SCDPC). Therefore, developing a method to evaluate the physicochemical changes under these conditions is crucial for accurately predicting the performance changes following BCP implantation.

In this research, we present an approach for assessing the physicochemical characteristics of BCP to precisely forecast the changes that take place within a cranial defect setting post-implantation. The validity of this assessment method was confirmed through experimental studies conducted under both *in vitro* and *in vivo* conditions. The performance of the BCP was analyzed before and after a six-month exposure period. To mimic a physiologically relevant *in vitro* cranial defect environment, pig blood and simulated body fluid were employed to closely reproduce the conditions at the implantation site, with controlled temperature and stable pH maintained throughout. Additionally, we analyzed and compared the characteristics of BCP exposed to both the* in vivo* environment and physiologically simulated *in vitro* cranial defect conditions to confirm their similarity. This research sought to estimate the degradation period of biodegradable medical devices by experimentally analyzing changes in their physicochemical properties after implantation, thereby reducing the reliance on clinical and animal studies. In contrast to earlier research, our study utilized pig blood to more precisely replicate the implantation site environment and centered on a medical device (BCP) rather than a scaffold. This study distinguishes itself from previous studies by conducting experiments over a six-month period.

In summary, our assessment of physicochemical property changes in BCP under the proposed SCDPC offers new perspectives for reliably forecasting post-implantation performance. This study contributes to the improvement of both the functionality and overall quality of biodegradable medical devices.

## Materials and methods

### Manufacturing of the 3D-printed medical implant

**Fig. 1:**
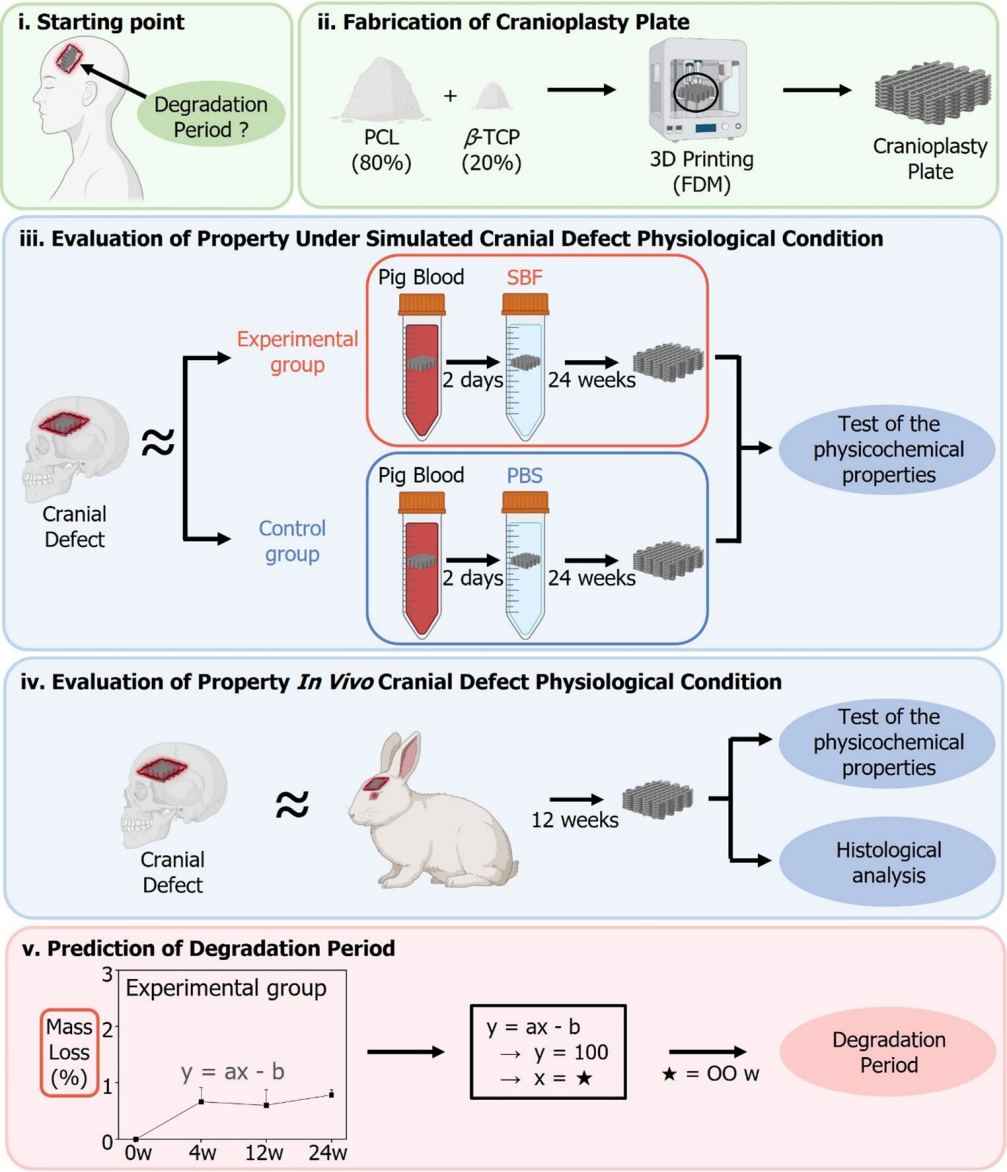
Schematic representation of this study. (i) Starting point of this study (ii) 3D printed PCL + *β*-TCP biodegradable cranioplasty plate (BCP) manufacturing process. (iii) A process to confirm and evaluate changes in each property of BCP exposed to simulated cranial defect physiological conditions (SCDPC) *in vitro*. (iv) A process to confirm and evaluate changes in the properties and histology of BCP exposed to cranial defect physiological conditions *in vivo*. (v) After plotting a graph of the rate of mass loss over time of BCP exposed to the experimental group, a process for predicting the degradation time of BCP after human implantation was derived using a linear equation

To manufacture medical devices using 3D printing, research grade polycaprolactone (M_w_: 50,000) (Polyscience, PA, USA) and beta-tricalcium phosphate (Thermo Scientific, NJ, USA) were used as raw materials. An 80:20 ratio of polycaprolactone (PCL) to beta-tricalcium phosphate (β-TCP) was used to fabricate BCP via fused deposition modeling (FDM) using a 3D printer (Rokit Healthcare, Seoul, Republic of Korea), followed by gamma-ray sterilization **(**Fig. 1**)**. Two types of test specimens were prepared based on the evaluation criteria: Type I (10 × 10 × 0.8 mm) for tests such as appearance, dimensions, porosity, mass, PCL content, and molecular weight, and Type II (10 × 30 × 0.8 mm) for tensile load testing. Prior to printing, the parameters were optimized to ensure that both the approved BCP and experimental specimens exhibited identical appearance and dimensions (Park et al. 2022). The detailed 3D printing settings were as follows: (1) Output method - Hotmelt, (2) Nozzle diameter − 0.4 mm, (3) Extrusion temperature − 108 °C, and (4) Printing speed − 4 mm/s. Gamma irradiation was employed to sterilize the 3D-printed BCP. Gamma sterilization was performed under the following conditions: (1) curing duration of 7 h, (2) radiation dose ranging from 15 to 30 kGy, and (3) a Sterilization Assurance Level (SAL) of 10⁻⁶.

### Establishment of a simulated cranial defect physiological condition

The BCP utilized in this research is a medical implant intended for cranial defect repair, designed to interface with blood and bodily fluids within the damaged cranial bone region. Accordingly, we sought to develop an SCDPC that accurately represents the physiological environment of cranial defects **(**Fig. 1**)**. To establish the SCDPC, an experimental group was prepared at 37 ± 1 °C and pH 7.4 ± 0.5, utilizing pig blood (Innovative Research, Novi, MI, USA) and calcium- and magnesium-containing simulated body fluid (Biochemazone, Alberta, Canada). To serve as a control, a group was prepared at 37 ± 1 °C and pH 7.4 ± 0.5 using pig blood (Innovative Research, Novi, MI, USA) and a phosphate buffer solution (Gendepot, Katy, TX, USA) containing sodium chloride and potassium phosphate. The BCP was submerged completely in each solution within a sealed tube to ensure full immersion. The specimens were incubated under stable temperature and pH conditions in two setups: (1) pig blood for 2 days followed by simulated body fluid (SBF) for 24 weeks, and (2) pig blood for 2 days followed by phosphate-buffered saline (PBS) for 24 weeks, using an incubator (Thermo Fisher Scientific, Waltham, MA, USA). The 2-day exposure duration in pig blood was selected to approximate the acute postoperative period following cranioplasty, during which subgaleal or epidural drains are typically maintained for approximately 48–72 h to manage postoperative bleeding (Oral et al. 2015; Choi et al. 2015). Plasma proteins such as albumin and fibrinogen rapidly adsorb onto hydrophobic PCL surfaces within this time window, initiating early hydrolytic chain scission and conditioning the implant interface for subsequent degradation (Mohan et al. 2019; Chen et al. 2022; Woodruff and Hutmacher 2010). Therefore, the 2-day exposure was chosen as a clinically representative early postoperative condition. Devices were retrieved at specific time points (4, 12, 20, and 24 weeks) for the assessment of their physicochemical characteristics.

### Analysis of physicochemical properties of devices under simulated cranial defect physiological conditions

After 24 weeks of exposure to SCDPC in both the experimental and control groups, changes in the physicochemical properties of BCP were evaluated. The evaluation included tests on (1) appearance, (2) dimensions, (3) porosity, (4) mass, (5) PCL content, (6) molecular weight, and (7) tensile load, with comparisons made between pre- and post-exposure conditions.

1. Appearance

Both visual inspection and microscopic analysis (ZEISS, Oberkochen, Germany) were conducted to identify any elements that might hinder the proper application of BCP. The general morphology of the specimen was documented by photographing the entire sample under a microscope. Microscopic magnification of the specimen’s central region was used to examine its microstructure and pore morphology. The three-dimensional shape of BCP is confirmed using a digital microscope (KEYENCE, Osaka, Japan). The cross-sectional structure is confirmed using micro-CT (Bruker, Billerica, MA, USA) scanner.

2) Dimensions

Specimens were imaged with a microscope (ZEISS, Oberkochen, Germany), and measurements of width, length, pore size, and line thickness were obtained using ImageJ software (NIH, Bethesda, MD, USA). A micrometer (Mitutoyo, Kawasaki, Japan) was used to measure the thickness at the center of each specimen.

3) Porosity

The specimen was aligned along its rotational axis, secured to a jig, and positioned within a micro-CT scanner (Bruker, Billerica, MA, USA). Following X-ray exposure, the acquired data were reconstructed into CT images, from which the specimen’s porosity was analyzed.

4) Mass

An analytical balance (Sartorius, Göttingen, Germany) was used to determine the mass of each specimen.

5) Content (PCL)

The PCL content of BCP was measured using a thermogravimetric analyzer (Mettler Toledo, Columbus, Ohio, USA). The specimen was positioned in a crucible, its initial mass recorded, then heated to a target temperature of 800 °C, and the resulting weight change was monitored.

6) Molecular weight

Following dissolution and pretreatment of the specimen in an appropriate solvent, a calibration curve was established using a standard material (Polystyrene; Resonac, Tokyo, Japan) via Gel Permeation Chromatography (Waters, Milford, MA, USA) (Ishiyama et al. 2024). The molecular weight of the specimen was subsequently determined relative to that standard.

7) Tensile load

A universal testing machine (Instron, Norfolk, MA, USA) was used to measure the maximum load at which the specimen fractured at its center. The testing parameters were based on ISO 527-1 and 527-3 standards for tensile testing of plastics, as well as R&D literature related to approved medical devices (Shim et al. 2017). Specific details include the use of a 100 N load cell for this experiment. The gap between the upper and lower grips was set to 10 mm, and the BCP was firmly fixed. While the lower grip remained stationary, the upper grip was pulled upward at a constant speed of 5 mm/min until the specimen fractured.

### Comparison of degradation behavior under simulated cranial defect conditions (*in vitro*) and actual implantation environments (*in vivo*)

Animal implantation studies were performed to compare the degradation behavior of BCP under SCDPC with that of BCP implanted in a living organism. This animal experiment (IACUC-2023-0083) received approval from the Institutional Animal Care and Use Committee (IACUC) of the Yonsei University Health System (Republic of Korea) and was carried out in accordance with its established guidelines. Animals were implanted into the cranial defect at 12 weeks of age using New Zealand White rabbits (females, 14 weeks old, weighing 2.4–2.9 kg). The animal study period was set at 12 weeks to allow for rabbit housing, growth, and the occurrence of adverse events. Twelve weeks post-implantation, performance evaluations and histological assessments were conducted. To assess performance, measurements of appearance, dimensions, mass, and molecular weight were carried out.

### Forecasting the degradation period of biodegradable polycaprolactone/β-tricalcium phosphate implants

A comparative analysis was conducted on the degradation behavior observed under SCDPC and actual *in vivo* implantation conditions. The time point at which a specific level of mass loss was reached was calculated, and a linear correlation between mass loss and exposure duration was established. To estimate the degradation period following human implantation, we calculated the time required for approximately 100% mass loss of the medical devices.

### Statistical analysis

To evaluate statistical differences among group means, one-way ANOVA was performed, followed by Duncan’s multiple range test for post-hoc analysis. A p-value of less than 0.05 (*p* < 0.05) was considered statistically significant. ANOVA analyses were conducted using R Studio software (Posit PBC, Boston, USA). For the datasets in Tables 1 and 2, paired t-tests were used to compare pre- and post-exposure values, with statistical significance similarly defined at *p* < 0.05. All t-test analyses were carried out using Microsoft Excel (Microsoft Corp., Redmond, WA, USA).

**Table 1:** Dimensions before and after exposure to an experimental group (pig blood (2 days) and simulated body fluid (24 weeks)) and a control group (pig blood (2 days) and phosphate buffer solution (24 weeks)). For statistical analysis, paired pre- and post-exposure results were analyzed using a t-test to determine significant differences between pre- and post-exposure. (*p* < 0.05)

Classification	Test items	Pre-exposure (mm)	Post-exposure (mm)	Rate-of-Change (%)	Statistical analysis
Control group	Width	10.439	10.310	−1.23	N.S.
	Length	10.454	10.388	−0.63	
	Thickness	0.8303	0.8300	−0.04	
	Pore	0.318	0.316	−0.52	
	Line Width	0.447	0.443	−0.86	
Experimental group	Width	10.414	10.335	−0.77	N.S.
	Length	10.164	10.153	−0.11	
	Thickness	0.876	0.876	0.00	
	Pore	0.349	0.350	0.28	
	Line Width	0.419	0.421	0.26	

**Table 2:** Physicochemical properties before and after exposure to an experimental group (pig blood (2 days) and simulated body fluid (24 weeks)) and a control group (pig blood (2 days) and phosphate buffer solution (24 weeks)). For statistical analysis, paired pre- and post-exposure results were analyzed using a t-test to determine significant differences between pre- and post-exposure. (*p* < 0.05)

Classification	Test items	Pre- exposure	Post- exposure	Rate-of-Change (%)	Statistical analysis
Control group	Porosity (%)	43.56	43.62	0.14	N.S.
	Mass (g)	0.0551	0.0546	−0.97	*p* < 0.05
	Content (PCL) (%)	80.72	82.03	1.61	N.S.
	Molecular Weight (kDa)	62,281	56,647	−9.05	N.S.
	Tensile Load (N)	27.90	9.64	−65.46	*p* < 0.05
Experimental group	Porosity (%)	43.95	44.10	0.34	N.S.
	Mass (g)	0.0550	0.0546	−0.79	*p* < 0.05
	Content (PCL) (%)	80.72	79.70	−1.27	N.S.
	Molecular Weight (kDa)	62,281	58,883	−5.46	N.S.
	Tensile Load (N)	27.90	8.57	−69.30	*p* < 0.05

## Results

### Assessment of physicochemical properties in medical devices subjected to simulated cranial defect physiological conditions

**Fig. 2:**
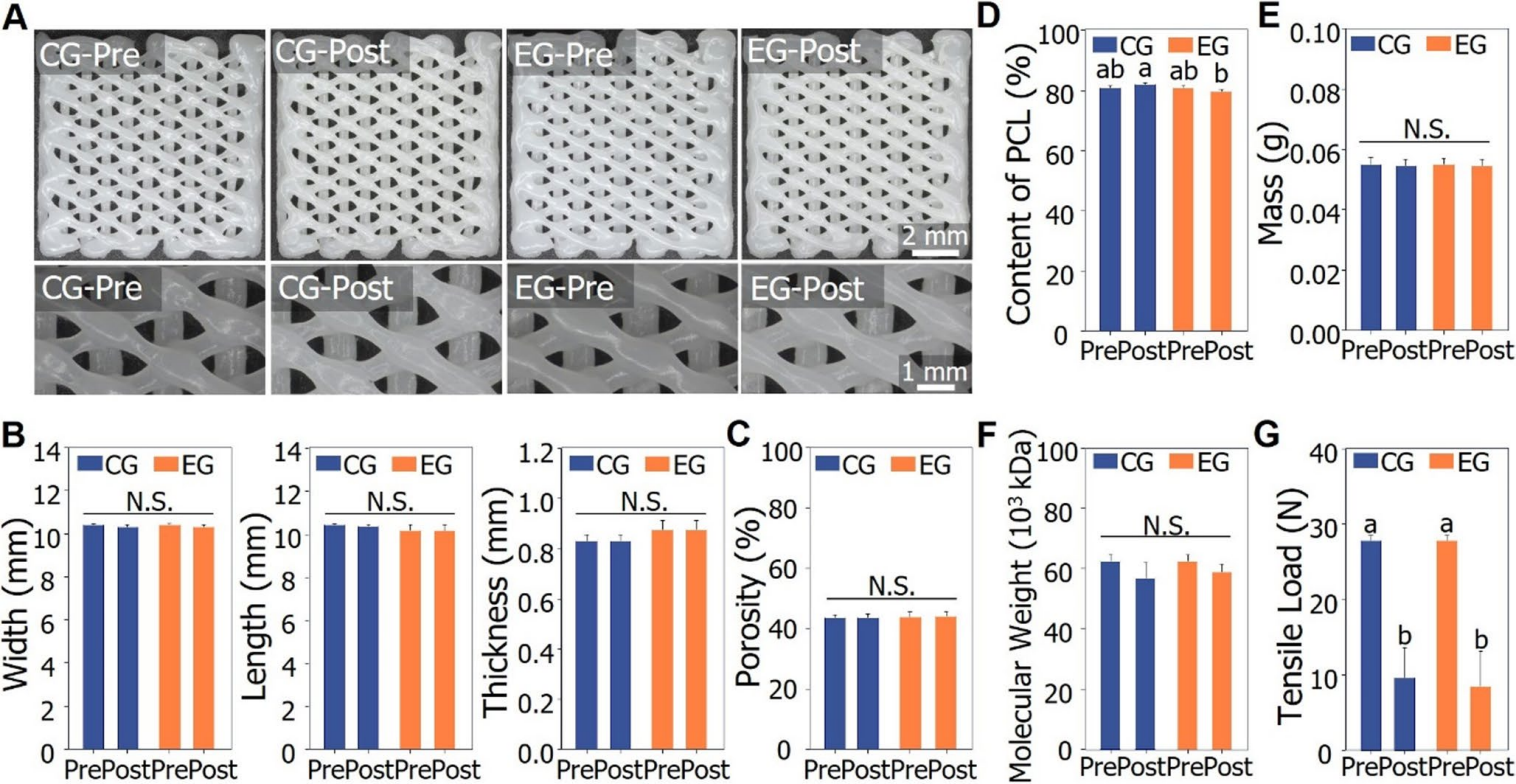
Physicochemical properties of BCP before and after exposure to an experimental group (EG) (pig blood (2 days) and simulated body fluid (24 weeks)) and a control group (CG) (pig blood (2 days) and phosphate buffer solution (24 weeks). (**A**) Overall appearance and detailed structure before and after exposure to CG (1st and 2nd from the left) and EG (3rd and 4th from the left). (**B**) Width (1st from left), length (2nd from left), and thickness (3rd from left) before and after exposure to CG and EG. (**C**) Porosity before and after exposure to CG and EG. (**D**) Content of PCL before and after exposure to CG and EG. (**E**) Mass before and after exposure to CG and EG. (**F**) Molecular weight before and after exposure to CG and EG. (**G**) Tensile load before and after exposure to CG and EG. (**B**–**G**) Statistical significance was analyzed using one-way ANOVA and Duncan’s multiple range test (*p* < 0.05). Graphs designated with different letters indicate that they are significantly different from each other

**Fig. 3:**
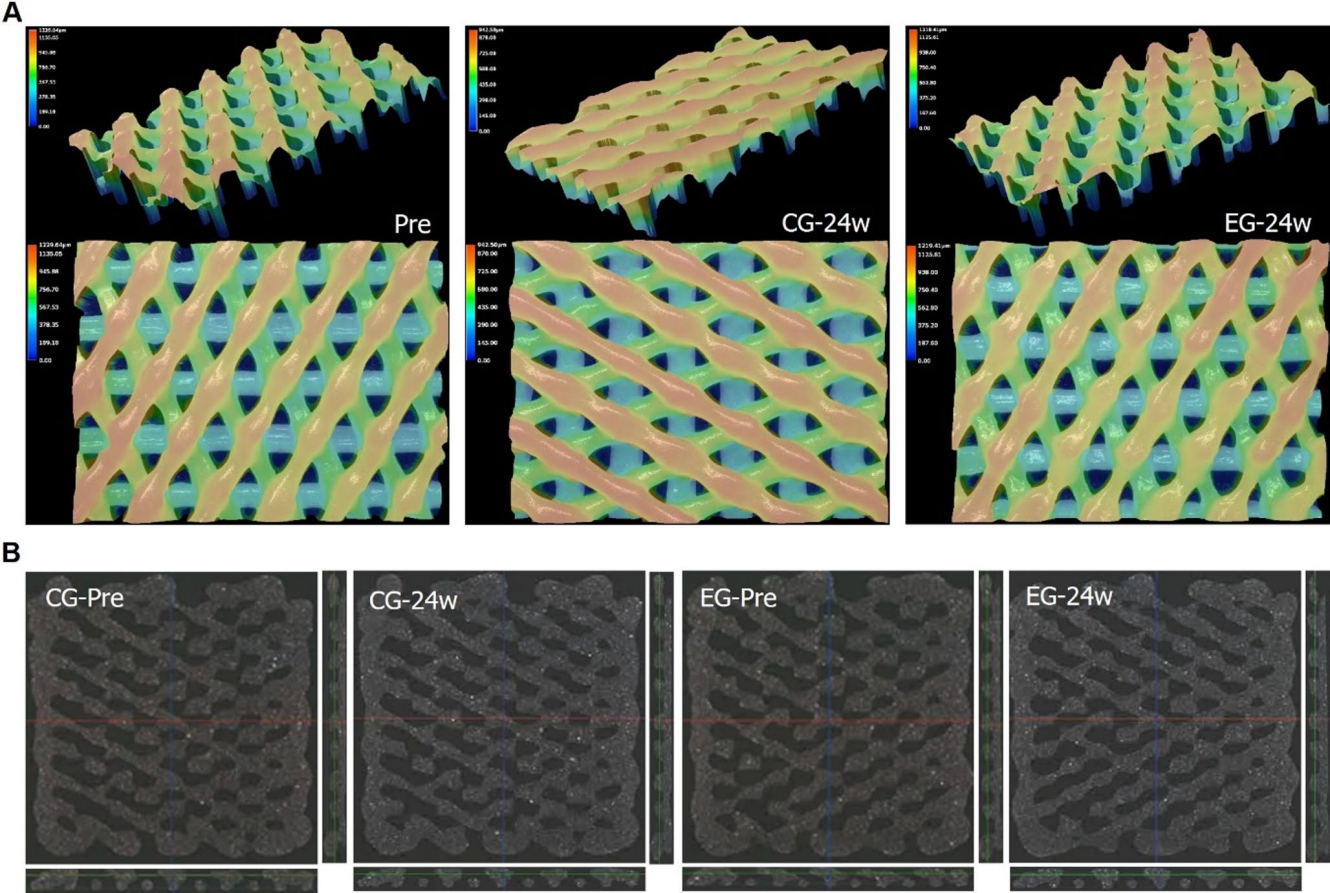
Analysis of BCP’s three-dimensional shape and cross-sectional structure. (**A**) Three-dimensional analysis of the morphology of BCP before exposure to simulated conditions, after 24 weeks of exposure to CG, and after 24 weeks of exposure to EG using a digital microscope. (**B**) Analysis of the cross-sectional structure of BCP before exposure to CG, after 24 weeks of CG exposure, before exposure to EG, and after 24 weeks of EG exposure using micro CT (Computed Tomography)

To assess the properties of biodegradable BCP in relation to the implantation site, two conditions were defined: an experimental group using pig blood and simulated body fluid (SBF), and a control group using pig blood and phosphate-buffered saline (PBS). After 24 weeks of exposure, the specimens were evaluated for appearance, dimensional stability, and porosity to assess their performance. To verify the degradation behavior, measurements of mass, PCL content, molecular weight, and tensile load were conducted. Visual inspections at 4, 12, 20, and 24 weeks revealed no noticeable differences in appearance between pre- and post-exposure specimens in both the experimental and control groups. Figure 2 A presents photographs of the full specimens and detailed regions following the 24-week exposure period, the longest duration assessed, to illustrate the external appearance test results. No substantial changes in the three-dimensional structure of BCP were observed among the specimens before exposure, after 24 weeks in PBS, and after 24 weeks in SBF **(**Fig. 3A**)**. Furthermore, the cross-sectional morphology remained largely unchanged before and after exposure in both the control group (PBS) and the experimental group (SBF), as shown in Fig. 3B. After 24 weeks of exposure, none of the measured dimensional parameters showed statistically significant differences **(**Table 1**).** Dimensional comparisons before and after exposure in both the control and experimental groups revealed no statistically significant variations **(**Fig. 2B**)**. Therefore, it was concluded that exposure to SCDPC did not result in any dimensional alterations. Porosity measurements showed a minor increase in both the experimental and control groups following 24 weeks of exposure; however, the difference was not statistically significant **(**Table 2**)**. Porosity comparisons between the control and experimental groups pre- and post-exposure revealed no statistically significant differences **(**Fig. 2C**)**. Hence, it was concluded that exposure to SCDPC did not lead to any significant alterations in porosity. PCL content analysis revealed a slight reduction in the experimental group after 24 weeks of exposure, though the change was not statistically significant in either the experimental or control group **(**Table 2**)**. The reduced PCL content indicates that BCP degradation occurred upon exposure to SCDPC. Mass measurements demonstrated a decrease in both the experimental and control groups after up to 24 weeks of exposure **(**Table 2**)**. According to the t-test, the control group showed a statistically significant mass reduction of − 0.97% after 24 weeks **(**Table 2**)**. In the control group, the mass changed by −0.61% at 4 weeks, −0.61 at 12 weeks, −0.85% at 20 weeks, and − 0.97% at 24 weeks. In the experimental group, a statistically significant change was observed after 24 weeks of exposure according to the t-test, with a mass reduction rate of − 0.79% **(**Table 2**)**. In the experimental group, the mass changed by −0.67% at 4 weeks, −0.61% at 12 weeks, −0.61% at 20 weeks, and − 0.79% at 24 weeks. Furthermore, ANOVA results comparing mass values before and after 24 weeks of exposure in both control and experimental groups revealed no significant differences across the four groups **(**Fig. 2E**)**. In both the experimental and control groups, mass reduction became more pronounced with increasing exposure duration. Therefore, it was confirmed that exposure to SCDPC leads to mass reduction in biodegradable medical devices due to degradation. Molecular weight testing demonstrated a downward trend in both the experimental and control groups after up to 24 weeks of exposure **(**Table 2**)**. However, the t-test results showed no statistically significant differences. In the control group, molecular weight decreased by − 4.65% at 4 weeks, − 5.64% at 12 weeks, − 8.16% at 20 weeks, and − 9.05% at 24 weeks. In the experimental group, the molecular weight was confirmed to change by −5.51% at 4 weeks, −4.73 at 12 weeks, −9.89% at 20 weeks, and − 5.46% at 24 weeks. ANOVA analysis comparing the four molecular weight data points before and after 24 weeks of exposure in both the control and experimental groups revealed no statistically significant differences **(**Fig. 2F**)**. When checking the changing trend of molecular weight, it was found that after a certain exposure period, the molecular weight tends to decrease. Thus, exposure to SCDPC was found to cause alterations in the chemical characteristics of biodegradable medical devices. Consequently, changes in molecular weight are considered to impact the mechanical behavior of these devices.

Tensile load testing revealed a significant decline in tensile strength in both the experimental and control groups following up to 24 weeks of exposure **(**Table 2**)**. In the control group, the tensile load changed by −5.20% at 4 weeks, −6.95% at 12 weeks, −21.88% at 20 weeks, and − 65.46% at 24 weeks. In the experimental group, the tensile load changed by −2.60% at 4 weeks, −16.21% at 12 weeks, −40.43% at 20 weeks, and − 69.30% at 24 weeks. Statistically significant reductions in tensile load were observed at 12, 20, and 24 weeks of exposure in both the experimental and control groups. Statistically significant differences in tensile load were observed between pre- and post-exposure measurements in both the control and experimental groups **(**Fig. 2G**)**. In both groups, prolonged exposure durations corresponded with greater reductions in tensile load. Thus, it was confirmed that exposure to SCDPC resulted in more pronounced alterations in the mechanical properties of biodegradable medical devices compared to other physicochemical characteristics. Consequently, the design and development of biodegradable medical devices requiring mechanical strength should account for the post-implantation decline in structural integrity. BCP samples exposed to both experimental and control conditions for up to 24 weeks exhibited no significant alterations in appearance, dimensions, or porosity **(**Fig. 2 A; Tables 1 and 2). In contrast, tensile load showed no Table reductions starting at 12 weeks, with the degree of decline progressively increasing over time.

### Comparison of physicochemical property assessment between simulated cranial conditions and *in vivo *implantation

An animal study was conducted to evaluate the degradation characteristics of biodegradable medical devices under conditions that simulate human implantation sites, by comparing *in vitro* SCDPC with actual *in vivo* implantation. BCP was implanted into cranial defects in rabbit skulls, and specimens were collected after 12 weeks to analyze degradation behavior and perform histological assessments. The results are outlined below:

1. Observation of common symptom

As a result of general symptom observation during the test period, minor abnormal symptoms, such as snuffles, were observed in some cases. Snuffles are infectious respiratory diseases with various causes, including climate change, infectious diseases, stress, and decreased immunity. Thus, it is considered unlikely that the observed abnormality was directly attributable to the test material.

2) Verification of *in vivo* degradation

**Fig. 4:**
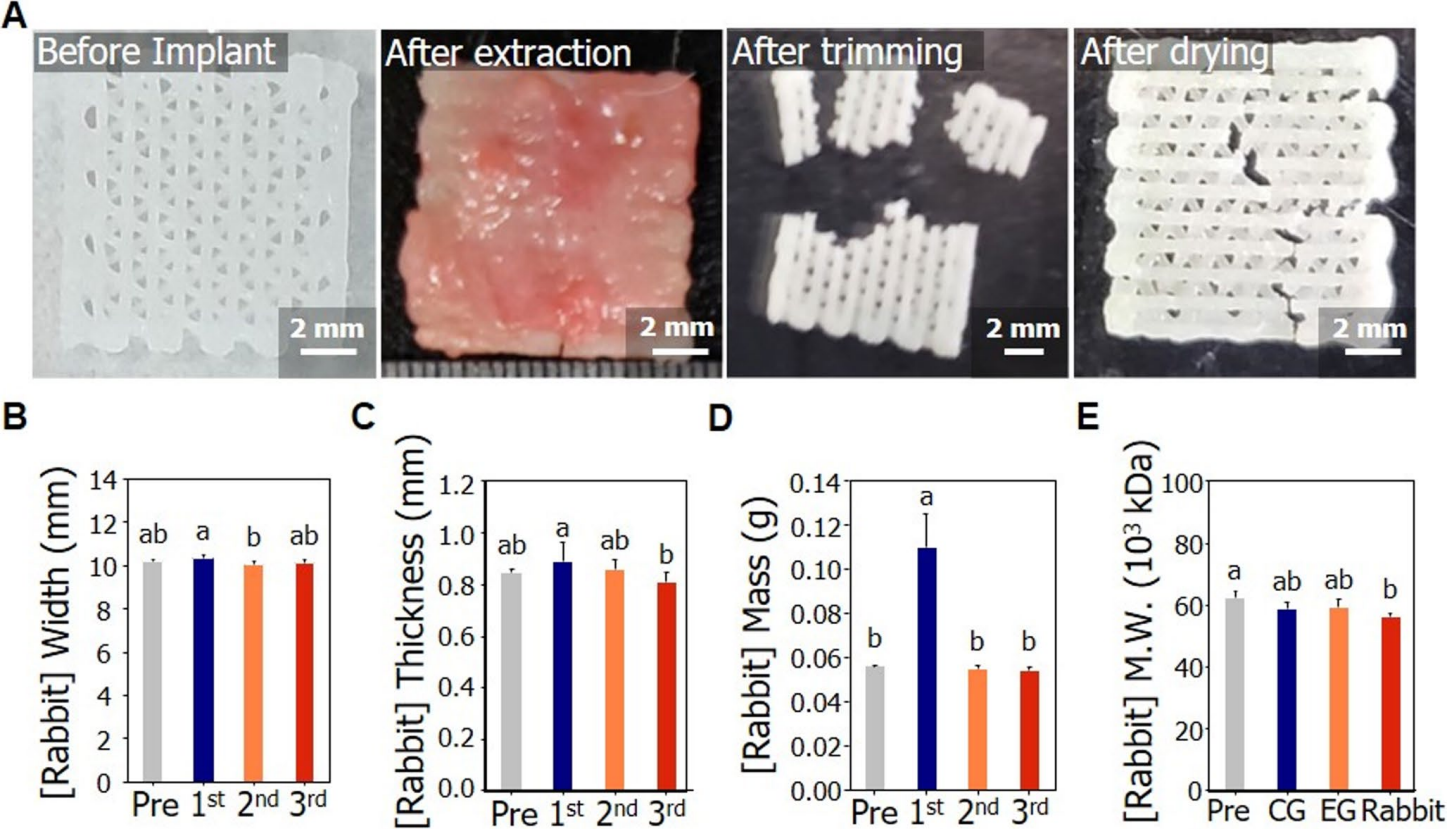
Confirmation of the physicochemical properties of the BCP *in vivo* implantation condition and an SCDPC (**A**) Appearance before implantation (1st from left) and after implantation (after extraction (2nd from left), after trimming (3rd from left), and after drying (4th from left)) in a rabbit at 12 weeks. (**B**) Width before (Pre) and after implantation (1st: after extraction, 2nd: after trimming, 3rd: after drying) in a rabbit at 12 weeks. (**C**) Thickness before (Pre) and after implantation (1st: after extraction, 2nd: after trimming, 3rd: after drying) in a rabbit at 12 weeks post-implantation. (**D**) Mass before (Pre) and after implantation (1st: after extraction, 2nd: after trimming, 3rd: after drying) in a rabbit at 12 weeks. (**E**) Comparative analysis of molecular weight after 12 weeks in four conditions: untreated, CG (pig blood (2 days) and phosphate buffer solution (24 weeks)), EG (pig blood (2 days) and simulated body fluid (24 weeks)), and rabbit. (**B**-**E**) Statistical significance was analyzed using one-way ANOVA and Duncan’s multiple range test (*p* < 0.05). Graphs designated with different letters indicate that they are significantly different from each other

The implanted specimens were examined and confirmed at the 12-week mark *in vivo*. The mass of the BCP was confirmed before implantation (Pre), immediately after extraction (1st), after trimming (2nd), and after drying (3rd) (Fig. 4A). The BCP, which was extracted 12 weeks after implantation, was surrounded by newly formed tissue, and its shape could not be determined. The tissue was removed and cleaned to check for masses after implantation. The sample was then dried for a more accurate mass measurement. Specimen damage was unavoidable during tissue removal. We found statistically significant differences in the masses of the animal specimens between the treatment steps (Fig. 4D). At 12 weeks, the specimen had a pre-implantation mass of 0.0563 ± 0.0003 g, increased to 0.1100 ± 0.0153 g upon extraction, then measured 0.0548 ± 0.0019 g after trimming, and 0.0546 ± 0.0014 g following final drying. Post-implantation BCP showed an increased mass compared to its pre-implantation state, likely due to tissue ingrowth within the pores and on the surface (Fig. 4D). After trimming and drying, the mass of the specimen was slightly lower than that before implantation. Nevertheless, there were no notable quantitative differences between the specimens before and after implantation.

3) Evaluation of histopathological characteristics

**Fig. 5:**
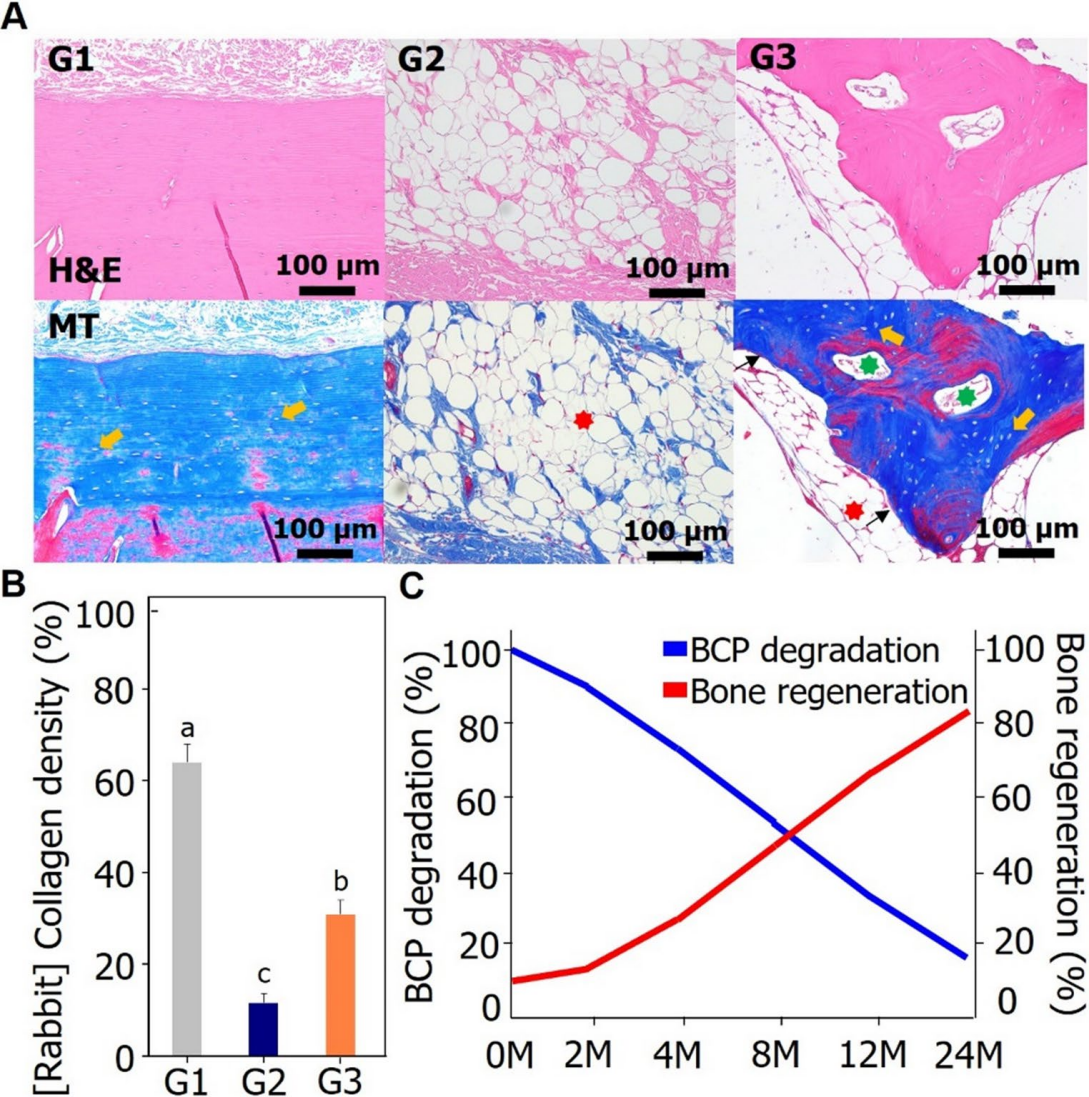
Results of *in vivo* implantation studies (12 weeks) to verify the effectiveness of BCP. (**A**) Representative H&E and Masson’ Trichrome (MT) staining histopathological profiles of skull (G1: normal control, G2-G3: calvarial bone defect induced group, G2: negative control (calvarial bone defect group), G3: test article (PCL/β-TCP implant group), Black arrow: osteoblast, Yellow arrow: osteocyte, Red star: bone marrow like tissue, and Green star: blood vessel). (**B**) Collagen density (G1: normal control, G2-G3: calvarial bone defect-induced group, G2: negative control (calvarial bone defect group), G3: test article (PCL/β-TCP implant group)). (**B**) Statistical significance was analyzed using one-way ANOVA and Duncan’s multiple range test (*p* < 0.05). Graphs designated with different letters indicate that they are significantly different from each other. (**C**) Schematic estimated timeline of BCP degradation and bone regeneration

Histological analysis of skull regeneration showed that the cranial defect group (G2) displayed disorganized collagen fibers, bone marrow, and hematoma—features typical of early bone regeneration—at the defect site, with no evidence of osteoblasts or osteocytes. In the test specimen (PCL and β-TCP) implanted group (G3), bone cells, blood vessels, osteoblasts, fat, and bone marrow were observed around the specimen (Fig. 5A). In particular, osteocytes were arranged in the form of osteons, bone units, and pseudobones, which were observed in the middle and late stages of bone regeneration (Fig. 5A). Histopathological examination (Masson’s trichrome) revealed significant differences in collagen density values among the normal group (G1), cranial defect group (G2), and test specimen (PCL and β-TCP) implantation group (G3) (Fig. 5B). The amount of collagen regenerated in the test specimen (PCL and β-TCP) implanted group (G3, 31.08%) was less than the normal group (G1, 64.06%), but more than the amount of collagen regenerated in the cranial defect group (G2, 11.80%). Among the two-cranial defect-induced groups (G2, G3), the bone regeneration of the cranial defect was more advanced in the test specimen (PCL and β-TCP) implanted group (G3).

4) Comparison of physicochemical properties between simulated cranial defect conditions and *in vivo* implantation

The evaluation and variation trends of physicochemical properties under SCDPC and animal implantation—both designed to reflect actual implantation site conditions—were comparatively analyzed. Twelve weeks after implantation into the damaged skull area of the rabbit, the skull implant was extracted and its width, thickness, and molecular weight were measured. The width and thickness of the BCP were measured before implantation (Pre), immediately after extraction (1st), after trimming (2nd), and after drying (3rd) (Fig. 4B and C). There was no statistical difference in the width before implantation (Pre) or after drying (3rd) (Fig. 4B). There was a statistical difference in the width immediately after extraction (1st) and after trimming (2nd) by ANOVA, but this was believed to be due to the regenerated tissue (Fig. 4B). There was a significant difference in thickness immediately after extraction (1st) and after drying (3rd) by ANOVA, but this was also believed to be due to the regenerated tissue (Fig. 4C). It is believed that the thickness was not that of the specimen itself.

We measured and compared the molecular weights to quantitatively confirm the physicochemical properties under *in vivo* implantation conditions. The molecular weight of the skull implant was measured under four conditions: (1) before exposure (Pre), (2) 12-week exposure in the control group (CG), (3) 12-week exposure in the experimental group (EG), and (4) 12-week implantation in the rabbits). The molecular weights in the CG, EG, and rabbit groups were lower than those before exposure. For the 12-week trial group, molecular weights and corresponding change rates were as follows: 62,281 pre-exposure, 56,258 after 12 weeks of animal implantation (− 9.67%), 59,337 after 12 weeks of exposure in the experimental group (EG; −4.73%), and 58,768 after 12 weeks in the control group (CG; −5.64%) (Fig. 4E). These results confirmed that the physicochemical reaction rate in the body was the fastest and the change was the greatest. For molecular weight, both SCDPC exposure and *in vivo* implantation led to reductions compared to pre-exposure values. The molecular weight loss *in vivo* was approximately twice that of SCDPC. This confirmed that the physiological response rate *in vivo* was faster than that in the SCDPC. This result validates that the SCDPC reasonably replicates *in vivo* conditions to a meaningful extent. These findings indicate that the degradation behavior of medical devices implanted in the human body can be approximated using simulated physiological environments.

### Prediction of degradation duration for polycaprolactone/β-tricalcium phosphate-based biodegradable implants

**Fig. 6:**
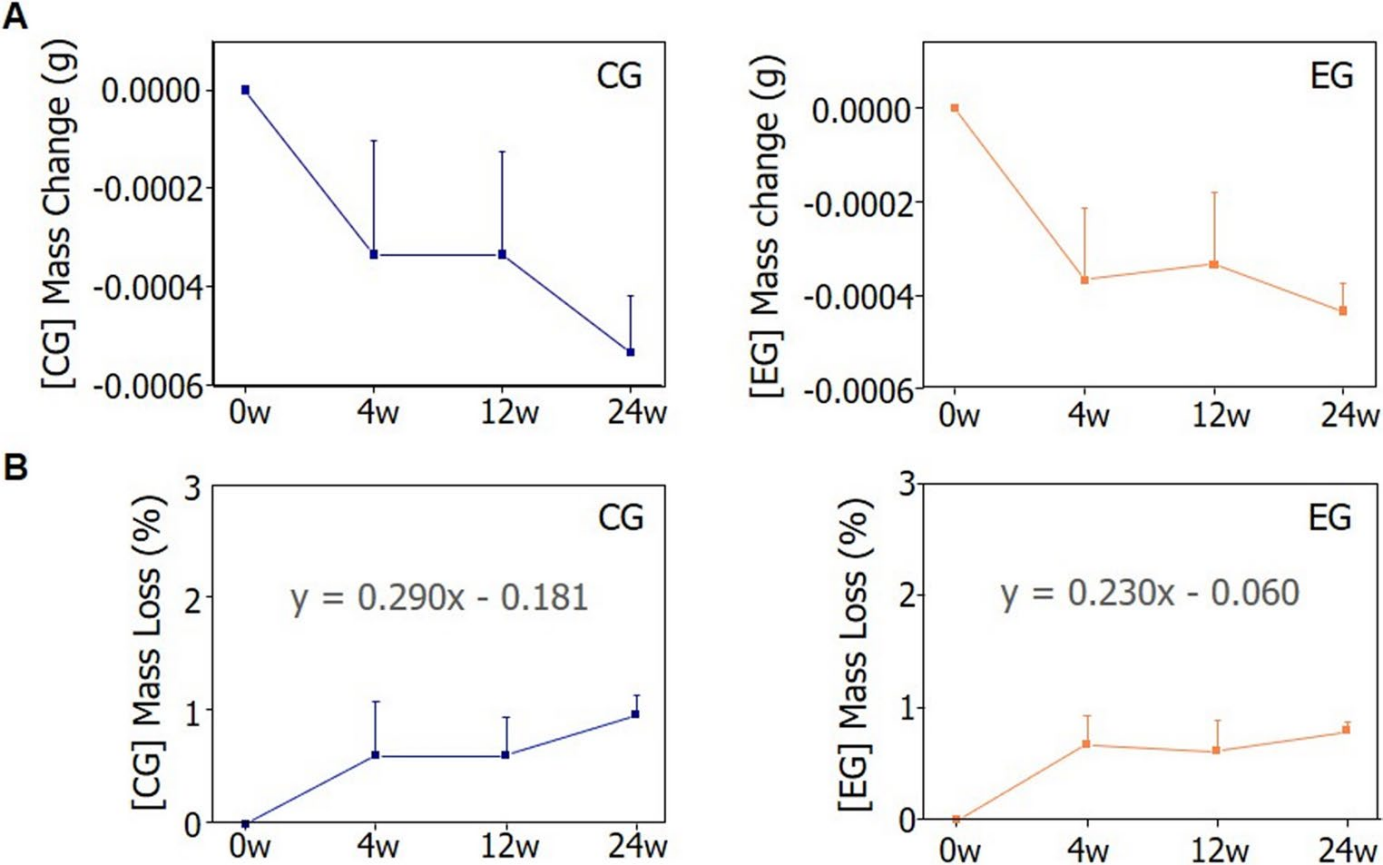
Confirmation of the mass reduction pattern according to the exposure period to the SCDPC. (**A**) (1st from left) Mass changes in BCP before CG exposure and after 4, 12, and 24 weeks of CG exposure. (2nd from left) Mass changes in BCP before EG exposure and after 4, 12, and 24 weeks of EG exposure. (**B**) Derivation of a linear equation to predict the degradation period of BCP after human implantation by analyzing the mass reduction pattern when exposed to CG and EG for 24 weeks

This study aimed to verify the degradation behavior of medical devices composed of polycaprolactone and beta-tricalcium phosphate—materials that degrade post-implantation—and to estimate their degradation period under human implantation conditions. Degradation behavior was assessed by tracking specimen mass over the SCDPC exposure and implantation periods, with the mass loss relationship represented by the equation in Fig. 6. Previous studies have indicated that there is typically a linear phase during which mass loss occurs proportionally over time, allowing the degradation period to be estimated using a linear equation based on mass reduction (Lam et al. 2008, 2009).

Specimen mass was measured at each time point, and a first-order linear equation (y = ax ± b) was derived to represent the correlation between the degradation period (x) and the mass reduction rate (y). The linear equation derived from the experimental group’s mass data was y = 0.230x − 0.060, with an R² value of 0.71 (Fig. 6B). For the control group, the mass data yielded a linear equation of y = 0.290x − 0.181 (Fig. 6B). The coefficient of determination (R²) for the equation was 0.87. Using a mass loss of 100% (y = 100) as the reference, the estimated time required for complete degradation in the experimental group was calculated. Using the derived linear Eq. 0.230x − 0.060, a mass loss rate (y) of 100 corresponds to an estimated degradation time (x) of approximately 435 days. We compared the degradation rates and volumes of the specimens with those of similar *in vivo* studies to derive a more accurate human body degradation period. *In vitro *testing demonstrated a mass loss of about 1% after six months of BCP exposure to SCDPC, aligning with prior* in vivo* findings that reported approximately 6% mass loss in PCL + TCP scaffolds implanted in rabbits over the same period (Lam et al. 2009). We found that the *in vivo* implant degraded approximately six times faster than SCDPC. We also found that the volume of BCP used in our experiments was 1.28 times larger than that of the scaffolds used in previous similar *in vivo* studies. Therefore, to estimate the degradation period under human body implantation conditions, considering the biodegradation rate and specimen size, 435 was divided by the biodegradation rate of 6 and multiplied by 1.28 to reflect the size of the specimen used in the *in vitro* experiment. Based on this calculation, the estimated *in vivo* degradation period for the experimental group was approximately 93 weeks (or 23 months), using the formula 93 = (435/6) × 1.28. When a 100% mass loss (y = 100) was applied to the linear equation, the time required for full degradation in the control group was determined. Applying a mass loss rate of 100 to the linear Eq. 0.290x − 0.181 yielded an estimated value of x ≈ 345 days. Taking into account the previously mentioned biodegradation rate and specimen dimensions, the estimated *in vivo* degradation period for the control group was approximately 74 weeks (around 18 months), calculated as 74 = (345/6) × 1.28.

This process enabled a comparative analysis of the projected degradation timelines for the medical devices under both conditions. According to the experimental group (EG) results, complete *in vivo* degradation of BCP is estimated to take about 93 weeks (approximately 23 months), while the control group (CG) results suggest a degradation period of around 74 weeks (approximately 18 months). Predicting the degradation timeline using SCDPC-based *in vitro* testing presents a viable alternative to *in vivo* studies and is expected to facilitate future research and development initiatives.

## Discussion

This study represents a significant advancement in the evaluation of BCP physicochemical properties by utilizing SCDPC to more accurately replicate *in vivo* degradation behavior (Fig. 1). This approach improves upon previous studies by utilizing pig blood, whereas earlier studies primarily relied on simpler methods, such as saline or PBS.

To accurately describe SCDPC, the temperature of the simulated environment was set to 37 ± 1 °C to reflect the bone temperature during implantation of the specialized cranial reconstruction material (Petrakis 1952). Additionally, the pH of the simulated environment was maintained at 7.4 ± 0.5, considering the physiological conditions of the blood, bone marrow, and bodily fluids in which the material is implanted (Nikolaeva 2018). Pig blood was incorporated based on the consideration that bleeding occurs for a period following BCP implantation, during which time the medical device is exposed to blood. In actual cranial surgery, cranial implants are inevitably exposed to blood immediately after surgery. We focused on creating *in vitro* conditions that closely mimic actual clinical situations and identifying and evaluating the resulting changes in physicochemical properties. Therefore, to maintain physiological relevance, we set the initial conditions to include pig blood. Using human plasma to simulate skull injury would provide the best human-like simulation. However, due to ethical concerns and practical difficulties in obtaining human plasma, we chose porcine blood, which is applicable in general *in vitro* experiments. Porcine blood was chosen because its plasma composition and physicochemical properties closely resemble those of human plasma and because pigs are widely used as a biomedical model (Lunney et al. 2021). Porcine blood is also frequently used in biomaterial testing (Christen and Vercesi 2013). Therefore, in our study, the devices were exposed to pig blood for the first two days. Future studies may explore different exposure periods to pig blood, such as 1 or 3 days, to better assess the effect of blood exposure duration on initial degradation patterns. Subsequently, to simulate exposure to body fluids, SBF was used in the experimental group, and PBS was used as the control group. Simulated body fluid (SBF), which closely resembles the composition of human blood plasma, is widely used in research to analyze bone behavior *in vitro*—particularly for assessing bone-bonding capability—as an alternative to *in vivo* evaluation of ceramic-based materials (Kokubo et al. 1990). Mechanical stimulation was excluded from the simulated environment, given that BCP is predominantly used in cranial applications. It can be reasonably inferred that direct mechanical stimulation from either internal or external forces is minimal or virtually nonexistent.

A 24-week exposure period was set to assess the property changes in BCP. Based on a review of the relevant literature, the experimental periods for the *in vitro* studies were set at 4, 12, and 24 weeks. Notably, a significant mass reduction was observed at approximately the 24-week mark. As a result, we acknowledge that the 24-week duration may be insufficient to comprehensively observe and understand the substantial degradation and property changes of the device intended for cranial defect implantation. This study was an initial attempt to simulate the environment of the BCP implant site *in vitro* and examine its effects. Therefore, we were unable to establish a long-term experimental period. Future research could be further developed by conducting longer *in vitro* and *in vivo* experiments or applying accelerated conditions to analyze long-term property changes and degradation periods. Furthermore, analyzing the stresses the skull experiences and establishing a simulated environment for *in vitro* experiments would allow for more accurate predictions of property changes and degradation periods. Furthermore, to verify the reliability of the simulated conditions, we aimed to assess the correlation between mass and molecular weight changes observed under SCDPC and those measured in *in vivo* implantation studies **(**Fig. 4D and E**)**. Therefore, maintaining a consistent experimental duration is essential for enabling valid comparisons between *in vitro* and *in vivo* results. However, extending *in vivo* studies beyond 12 weeks is difficult due to practical constraints, including animal growth and health considerations. As a result, this study implemented a 24-week *in vitro* protocol alongside a 12-week *in vivo* experiment. To assess degradation beyond the 24-week timeframe, we suggested predicting the required degradation period by analyzing the mass loss trend within that duration. Although long-term *in vitro* and *in vivo* studies over two years would provide the most precise evaluation of degradation and performance in long-lasting medical devices, such an approach is impractical given the time constraints associated with medical device development. Thus, based on previous studies and the practical constraints of animal testing, we determined that a 24-week period for *in vitro* experiments and a 12-week period for* in vivo* experiments were reasonable and pragmatic approaches in the present study. The 12-week *in vivo* implantation period was chosen because tissue growth in calvarial animal models is significantly advanced at 24 weeks, making specimen collection and measurement difficult. Therefore, we conducted a 12-week experiment. After 12 weeks, both the molecular weight in the rabbit experiment and the 12-week SCDPC experiment decreased compared to before exposure to *in vitro* and *in vivo* conditions. This suggests that *in vivo* and SCDPC conditions are somewhat similar. Therefore, we concluded that degradation and changes in physicochemical properties occur starting at 12 weeks under both *in vitro* and *in vivo* conditions. Based on the results from the 24-week SCDPC experiment, we estimated a correlation with long-term degradation.

We examined the appearance of BCP under a microscope to confirm the degradation of the BCP after 24 weeks of exposure to SCDPC. Unfortunately, no changes in appearance were observed after 24 weeks of exposure, sufficient to confirm the degradation. It is believed that a longer-term experiment would be necessary to confirm the degradation. Following 24 weeks of SCDPC exposure, BCP showed no notable alterations in external morphology, size, or porosity **(**Fig. 2 A, B and C**)**. These findings imply that external morphology and structural features may not serve as reliable indicators for evaluating post-implantation changes in the physicochemical properties of BCP. In contrast, physicochemical analysis after 24 weeks of SCDPC exposure showed a marked reduction in both mass and tensile strength of BCP **(**Fig. 2E and G; Table 2**)**. This decrease is likely attributable to the degradation of PCL, which is used in the preparation of BCP, thereby affecting its physicochemical properties. PCL degradation under SCDPC and* in vivo* conditions is dominated by bulk hydrolysis, with initial chain scission occurring in amorphous regions (Woodruff and Hutmacher 2010; Christen and Vercesi 2020). Enzymatic activity from blood-derived esterases may contribute to accelerating early degradation (Peng et al. 2010). Although acidic oligomers such as 6-hydroxycaproic acid are generated, physiological buffering minimizes large pH shifts, leading to a hydrolysis-driven degradation profile (Woodruff and Hutmacher 2010). Polycaprolactone (PCL) degrades through hydrolysis in aqueous environments, and thus degradation is anticipated in both PBS and SBF conditions. After 24 weeks of exposure, the experimental group exhibited a statistically significant mass decrease of roughly − 0.79% compared to the baseline **(**Table 2**)**. The control group exhibited a comparable pattern, showing a mass decrease of about − 0.97% over the same 24-week period **(**Table 2**)**. Nonetheless, no statistically significant alterations in molecular weight or PCL content were detected in BCP specimens exposed to either SCDPC condition **(**Fig. 2D and F; Table 2**)**. While there were numerical changes in molecular weight and PCL content due to PCL degradation after SCDPC exposure, no significant changes were observed. This may be due to measurement variability or sensitivity. Therefore, further research using multi-detector GPC and DMA for viscoelastic properties would yield more precise results for more precise detection of degradation in the early stages. These results should allow us to infer the correlation between changes in molecular weight and PCL content and early degradation. Conversely, tensile load testing revealed a pronounced decline in BCP’s mechanical properties over 24 weeks, with reductions of − 69.30% in the experimental group and − 65.46% in the control group **(**Table 2**)**. Notably, the tensile load exhibited a more pronounced decline in both groups **(**Fig. 2G**)**. No statistically significant differences were identified between the control and experimental groups. It is hypothesized that thermal stress is the primary factor influencing PCL degradation because the thermal conditions were identically controlled in both groups. Because the key difference between SBF and PBS lies in the presence of certain inorganic salts that do not cause enzymatic reactions, variations in composition are unlikely to influence post-implantation degradation behavior. Through tensile loading tests, we reliably confirmed the significant degradation in the mechanical properties of BCP following exposure to SCDPC.

We implanted BCP in rabbits for 12 weeks to compare its physicochemical properties *in vivo* with those observed in SCDPC. The general morphology and microstructure of the implanted BCP closely resembled those observed under SCDPC conditions, with no clinically significant alterations detected in the rabbit-implanted specimens. Nonetheless, evident tissue growth was observed on the BCP surface as well as within its pores **(**Fig. 4A**)**. We aimed to evaluate mass loss resulting from BCP degradation after implantation in rabbits; however, tissue formation on the BCP hindered precise mass measurement. After harvesting the rabbit specimens, tissues were manually removed using tweezers, washed, and dried. The measured mass decreased slightly; however, this reduction was not statistically significant **(**Fig. 4D**)**. To further evaluate degradation, we analyzed the molecular weight of BCP after implantation in animals and compared it with that of BCP under other conditions. In the* in vivo* rabbit experiment, mass measurement was difficult due to tissue regeneration and other factors, making it impossible to quantitatively confirm and compare changes in specimen mass over the implantation period. However, unlike mass, molecular weight could be quantitatively confirmed using analytical equipment. Furthermore, both the *in vivo* rabbit experiment (12 weeks) and the SCDPC experiment (12 weeks) showed a slight decrease in molecular weight compared to pre-exposure. Therefore, it is reasonable to conclude that the molecular weight of BCP decreases both *in vivo* and *in vitro*, independent of the change in mass. The results indicated that the molecular weight decreased more rapidly in rabbits over 12 weeks (−9.67%) **(**Fig. 4E**)** than in SCDPC (experimental group: −4.73%, control group: −5.64%). This suggests that the degradation rate *in vivo* tends to be higher than that *in vitro*. Therefore, SCDPC can serve as an appropriate model for predicting degradation in clinical applications, particularly when a complementary coefficient is introduced to account for differences in the reaction rates between the two environments. In this case, the complementary coefficient was estimated to be 2, indicating that the degradation of molecular weight occurs approximately twice as fast *in vivo* as *in vitro*. However, the precise determination of this coefficient requires further experimentation with rigorous control of variables to ensure consistency and accuracy. Statistical analysis revealed no significant changes in the width or thickness of BCP between pre- and post-implantation measurements **(**Fig. 4B and C**)**. In this study, rabbits were selected as the animal model to assess the extent to which SCDPC replicates *in vivo* conditions, with BCP implants directly inserted into cranial defects to evaluate their behavior in a physiologically relevant environment.

Through the assessment of physicochemical property changes in BCP following SCDPC exposure, we inferred *in vivo* degradation patterns by analyzing *in vitro* results and applying correction factors. The results showed that mass loss increased proportionally with the duration of SCDPC exposure, enabling the development of a correlation equation linking exposure time to mass reduction **(**Fig. 6B**)**. This equation served as the basis for estimating the *in vivo* degradation timeframe of BCP. Mass was selected as the primary indicator for estimating the degradation period based on the following rationale. Mass reduction, a direct consequence of polymer degradation, is considered the most fundamental and dependable indicator of biodegradable polymer breakdown. Although the mechanical strength and porosity may be secondary factors influenced by polymer degradation, mass reduction is the most direct and quantifiable measure. Accordingly, this study concentrated on tracking mass loss over time as the primary means of estimating the *in vivo* degradation period. Based on the equations derived in this study, the projected degradation periods of BCP under SCDPC conditions were 435 weeks for the experimental group and 345 weeks for the control group **(**Fig. 6B**)**. Referring to a previous study by Lam, Hutmacher, Schantz, Woodruff, and Teoh (Lam et al. 2009), we estimated the *in vivo* degradation time to be 93 weeks for the experimental group and 74 weeks for the control group, based on the degradation rate and specimen size. The BCP used in this study was identical to the scaffold in the literature in terms of raw materials and porous structure. Therefore, we determined that it was similar to the specimens we tested. To estimate more accurately the degradation time, we incorporated the degradation rate (6) and specimen volume (1.28) from the literature into our estimates. Degradation period predictions are directly affected by changes in physical and mechanical properties, such as mass and tensile load. Therefore, factors such as external shape and size will ultimately have a primary impact on degradation. Therefore, to more accurately estimate degradation period, we analyzed and incorporated the specimen size and volume from both this study and the reference study. In summary, we suggest that SCDPC serves as a feasible model for estimating the degradation timeline of BCP after human implantation, assuming a precise correction coefficient is applied.

Histopathological analysis revealed distinct differences in bone regeneration patterns among the experimental groups **(**Fig. 5A and B**)**. In the cranial defect group (G2), early stage bone regeneration was observed, characterized by irregularly arranged collagen tissue, bone marrow, and hematoma, with no osteoblasts or osteocytes. By contrast, the test specimen implantation group (G3) demonstrated more advanced bone regeneration in the presence of bone cells, blood vessels, osteoblasts, fat, and bone marrow surrounding the implanted material. Notably, the osteocytes were arranged in an osteon-like structure and showed pseudobone formation, indicating mid-to-late-stage bone regeneration. In this study, we first aimed to evaluate changes in physicochemical properties after implantation and determine the resulting degradation patterns. While bone regeneration was confirmed through *in vivo* experiments in rabbits, the relationship between BCP degradation and new bone formation was not fully confirmed, a limitation. Therefore, in the future, we believe that quantitatively analyzing bone formation using micro-CT and analyzing its correlation with degradation will allow us to elucidate how the degradation rate relates to the bone healing process. Masson’s trichrome staining further confirmed the differences in collagen regeneration between the groups. Collagen density in the implanted group (G3) was markedly greater than in the cranial defect group (G2), yet remained lower than that observed in the normal bone group (G1). This suggests that while bone regeneration in G3 was not yet fully restored to normal levels, implantation of PCL and β-TCP significantly enhanced bone healing compared with untreated defects. These findings support the potential of BCP-based materials to promote bone regeneration in cranial defects.

This discussion underscores the importance of tailored design strategies that consider the progressive decline in mechanical properties over time. For example, increasing cross-linking density or incorporating protective coatings may reduce tensile strength degradation, contributing to improved long-term stability. Moreover, developing an internationally standardized SCDPC-based testing method for assessing post-implantation physicochemical properties could greatly minimize reliance on animal testing. To facilitate the standardization of SCDPC in a future regulatory context, several protocol elements should be clearly defined. These elements include (1) specifying the blood source and age, (2) the applied anticoagulation method and blood concentration, (3) standardized temperature, pH, and exposure time during the blood contact phase, (4) the composition, temperature, pH, and exposure time of the SBF used after blood contact, and (5) the type and intensity of mechanical stimulation comparable to the load applied to the skull. Establishing these parameters will improve interlaboratory reproducibility and enable SCDPC to serve as a more robust and regulatory-relevant human-simulating *in vitro* experimental evaluation platform for assessing degradation behavior.

## Conclusion

This study developed an SCDPC model that effectively predicts the *in vivo* degradation behavior of BCP with high reliability. The consistency between degradation outcomes observed in SCDPC and *in vivo* experiments underscores the reliability and precision of this approach. In summary, this research demonstrates that SCDPC is a reliable method for assessing the post-implantation degradation and performance of biodegradable medical devices, while also contributing meaningful insights to both scientific understanding and practical implementation. Future investigations should build upon these findings by examining diverse polymers and composite materials, conducting longer-term studies, and incorporating computational modeling to enhance prediction accuracy.

## Data Availability

Data will be made available on request.
